# Stepping and tapping: combining motor tasks improves cognitive classification

**DOI:** 10.1007/s11357-025-01678-7

**Published:** 2025-05-08

**Authors:** Kaylee D. Rudd, Michele L. Callisaya, Katherine Lawler, Alastair J. Noyce, James C. Vickers, Jane Alty

**Affiliations:** 1 Wicking Dementia Research and Education Centre, University of Tasmania, 17 Liverpool Street, Hobart, TAS 7000 Australia; 2 Medical Science Precinct, Menzies Institute for Medical Research, University of Tasmania, Hobart, TAS Australia; 3 Peninsula Clinical School, Monash University, Frankston, VIC Australia; 4 School of Allied Health, Human Services and Sport, La Trobe University, Bundoora, VIC Australia; 5 Centre for Preventive Neurology, Wolfson Institute of Population Health, Mary University of London, London, Queen UK; 6 School of Medicine, University of Tasmania, Hobart, TAS Australia; 7 Royal Hobart Hospital, Hobart, TAS Australia

**Keywords:** Dementia, Mild cognitive impairment, Subjective cognitive impairment, Gait, Digital biomarker, ISLAND

## Abstract

Gait and key-tapping are individually associated with mild cognitive impairment (MCI) and dementia. However, it is unclear if these motor functions are correlated, or whether combining them improves classification of objective (dementia, MCI) and subjective cognitive impairment (SCI). We recruited 73 participants with dementia, 106 MCI, 57 SCI, and 83 cognitively healthy controls (HC). Consensus diagnosis was made after gold-standard interdisciplinary assessment. Fast-paced gait was assessed on an electronic walkway and fast-paced key-tapping on a computer keyboard. Correlations between gait and key-tapping measures (speed, frequency, variability and contact) were tested using Pearson’s correlation. Classification accuracy was calculated using area under receiver-operating-characteristic curves (AUC) and compared to the null model comprising age, sex and education. Gait and key-tapping measures correlated moderately. Combined gait and key-tapping speed improved classification accuracy of dementia (.97), and MCI (.91), from HC, but not SCI, compared to gait (dementia: .94, MCI: .87) or the null model (dementia: .89, MCI: .79). Gait and key-tapping measures were associated with Alzheimer’s disease and vascular dementia, but the effect size for key-tapping variability was larger in vascular dementia (*β*: 225.71) compared to Alzheimer’s disease (*β*: 38.30). Gait and key-tapping variability was associated with non-amnestic MCI. Measures of gait were correlated with corresponding key-tapping measures, but their association with cognitive impairment was not the same. Combining gait and key-tapping motor measures improved classification accuracy of MCI and dementia. This suggests gait and key-tapping measures provide information about different aspects of motor-cognitive association worth further investigation.

**Supplementary Information:**

The online version contains supplementary material available at 10.1007/s11357-025-01678-7.

## Introduction

Dementia prevalence is surging and projected to reach 150 million globally by 2050 [1]. Risk modification may prevent or delay 45% of cases and, to facilitate effective targeted prevention strategies, there is a need to detect at-risk groups [2]. Those with subjective cognitive impairment (SCI) and mild cognitive impairment (MCI) are at increased risk for developing dementia in the future [3]. Over the last 30 years, a growing body of evidence found changes in motor function occurs at these early stages of the dementia continuum [4–8]. Most motor-cognitive function research has evaluated gait; cross-sectional studies found that subtle changes, such as slower speed and greater variability of stepping, are associated with MCI and dementia [9–12] and longitudinal studies found gait changes precede dementia by decades [6, 13–15]. Additionally, gait measures are associated with dementia-related brain imaging markers such as hippocampal volume [16, 17]. Thus, gait is recognised as an established biomarker of cognitive impairment [18].

Other motor functions have been far less explored across the dementia continuum, but new cross-sectional evidence shows impaired upper limb motor function (ULMF), such as slowed speed and weaker grip strength, are also associated with subtle cognitive impairments, MCI and dementia [15, 19, 20]. In our previous studies, we found that key-tapping improved the estimation of cognitive function in cognitively asymptomatic community-dwellers as well as in MCI and dementia [19, 21]. The association of key-tapping measures with dementia-related brain imaging markers has not been investigated extensively, but one study (2023) found key-tapping variables were associated with reduced hippocampal volume [22].

Whilst measures of gait and key-tapping are known to individually improve classification of cognitive impairment [9, 11, 19, 22–24], they have rarely been evaluated in combination. Combining information from different sources provides a more precise and comprehensive view of a disease, especially aspects that may be otherwise absent or overlooked [25]. Findings of two previous studies on a combination of upper and lower limb measures suggest superiority of combined models over individual upper or lower limb measures [15, 26], although only one of these studies had an MCI group (*n*: 45) and neither included an SCI group.

Furthermore, there has been no previous investigation of how measures of gait function, such as stepping frequency, relate to conceptually similar measures of hand motor function, such as tapping frequency. Thus, there remains a need to further examine whether combining upper and lower limb motor measures improves classification of cognitive impairment; this presents an opportunity for early non-invasive detection of dementia risk as well as deepening our understanding of motor-cognitive associations.

This study aimed to determine (1) whether gait and key-tapping measures are individually associated with cognitively healthy, SCI, MCI and dementia groups; (2) the correlations between gait measures and key-tapping measures, and (3) whether combining gait and key-tapping measures increases classification accuracy of cognitive diagnoses over and above a model comprising age, sex and years of education (hereon referred to as the Null Model) which are known risk factors for cognitive impairment [2, 27]. We hypothesised that selected motor measures would be associated with cognitive diagnoses; that gait measures would be correlated with key-tapping measures; that gait and key-tapping would have similar classification accuracy and, when combined, would improve classification accuracy of cognitive diagnoses.

## Methods

### Participants and recruitment

We recruited consecutive participants from the Island Study Linking Ageing and Neurodegenerative Disease (ISLAND) Cognitive Clinic at the University of Tasmania, Australia. The Clinic provides a gold-standard interdisciplinary consensus diagnosis for adults with more than 3 months of cognitive symptoms based on comprehensive assessments comprising medical, neuropsychological and neuroimaging; the detailed protocol has been published [28, 29]. All participants attending the Clinic had their capacity to consent to research formally assessed by a trained clinician [28]. Consenting participants with a diagnosis of dementia (Alzheimer’s disease (AD), vascular dementia (VaD) or mixed AD and VaD dementia), MCI (grouped by amnestic (aMCI) and non-amnestic (nMCI) subtypes), and SCI were recruited. For MCI subtypes, those performing poorly in tests of the memory domain, with or without involvement of other domains, were categorised in the aMCI group. The nMCI group comprised those who performed poorly on tests of cognitive domain(s) other than memory, such as language, attention or executive function [30].

Cognitively healthy control (HC) participants were recruited from the ISLAND Project, a community-based longitudinal dementia risk reduction study of adults aged ≥ 50 years old with a published protocol [31]. HCs with an Addenbrooke’s Cognitive Examination-version three (ACE-III) score ≥ 95/100 and no reported subjective or objective cognitive impairment were included in the analysis. This study was approved by the University of Tasmania Human Research Ethics Committee [ref. H0018639] and all participants provided written consent.

### Data collection

All motor and cognitive tests were completed on a single day during an in-person visit to the Clinic [28]. Demographic data included age (years), sex (male/female/other/prefer not to say), full-time equivalent years of education (hereon referred to as education), hand dominance (self-reported), height and Body Mass Index (BMI). BMI was measured for its effect on risk of developing dementia and cardiovascular diseases in mid- and late-life [2, 32]. All participants completed ACE-III, the Trail Making Test parts A and B and questionnaires on mood (Hospital Anxiety and Depression Scale [HADS] for HC; Geriatric Depression Scale [GDS] for Clinic participants).

### Gait assessment

Gait assessments were completed on a 6 m electronic, pressure sensor embedded walkway (Zeno™ Gait Analysis System, ProtoKinetics LLC, Havertown PA. Scan rate: 120 Hz, Spatial resolution: 1.27 cm). Data from the gait mat were processed using ProtoKinetics Movement Analysis Software (PKMAS) version 5.09c, ZenoMetrics, LLC, USA [https://protokinetics.com/zeno-walkway/]. The start and return lines on the floor were marked with tape at 2 m from either end of the walkway to allow for acceleration/deceleration [33]. Participants completed two consecutive passes on the gait mat walking being instructed to walk as fast as they could without running (fast-paced walk).

### Key-tapping assessment

Upper limb motor assessments were completed using the online BRadykinesia Akinesia INcoordination (BRAIN) test that is a validated motor test in Parkinson’s disease [19, 34]; participants were instructed to use the index finger of one hand to alternately tap two computer keys 15 cm apart quickly and accurately for 30 s, with left hand assessed first.

### Selection of motor variables for analysis

To analyse motor function of upper and lower limbs, we selected four features that have been commonly used in previous studies [9, 13, 19, 22, 35, 36]: speed, frequency, variability and the contact time of a body part with a surface (hereon referred to as contact). For gait analysis, these measures were (1) speed, (2) cadence (number of steps/min) — hereon referred to as frequency, (3) variability (coefficient of variation of step time) and (4) contact (stance time; mean time (seconds) with a foot in contact with the gait mat). The respective key-tapping variables were (1) speed (mean inter-tap speed), (2) frequency (number of key-taps/30 s), (3) variability (coefficient of variation of mean key-tap time travelling between keys) and (4) contact (mean time (milliseconds) spent with a fingertip depressing a key). See Supplementary Table 1.

### Statistical analysis

Between cognitive group differences were examined using an ANOVA. Normal distribution of variables was tested using P-P plots. To determine the association between each of the gait and key-tapping variables (independent variable) and cognitive diagnosis (dependent variable), we used linear regression in a model adjusted for potential confounding variables of age, sex and education. To determine the associations between gait and key-tapping measures, we first examined their unadjusted correlations using Pearsons’s correlations. Adjusted associations were then examined using linear regression analysis with age, sex and education as covariates. We tested the participants’ height as a potential confounder for motor variables. However, as adding height to regression models of gait and key-tapping variables caused < 10% change in the coefficient, it was not included [37]. We also tested the interaction between cognitive diagnosis and each key-tapping variable (key-tapping variable × cognitive diagnosis) with each gait measure as the outcome variable.

To determine whether key-tapping measures increase classification accuracy of cognitive diagnoses, we first examined the classification accuracy of Model One — comprising the Null Model variables (age, sex and education) plus an individual gait variable using the area under receiver operating characteristic (ROC) curves (AUC). In Model Two, we also included key-tapping measures and calculated the AUC. We rejected the null hypothesis, that key-tapping variables do not improve classification over and above gait and confounding variables, if there was a significant difference between AUC of Models One and Two. The significance level was set at *P* < 0.05, the *P*-value utilised by studies of similar structure and exploratory design [13, 15, 26, 38]. Statistical analysis was completed using STATA 18.0.

## Results

### Characteristics of participants

**Fig. 1:**
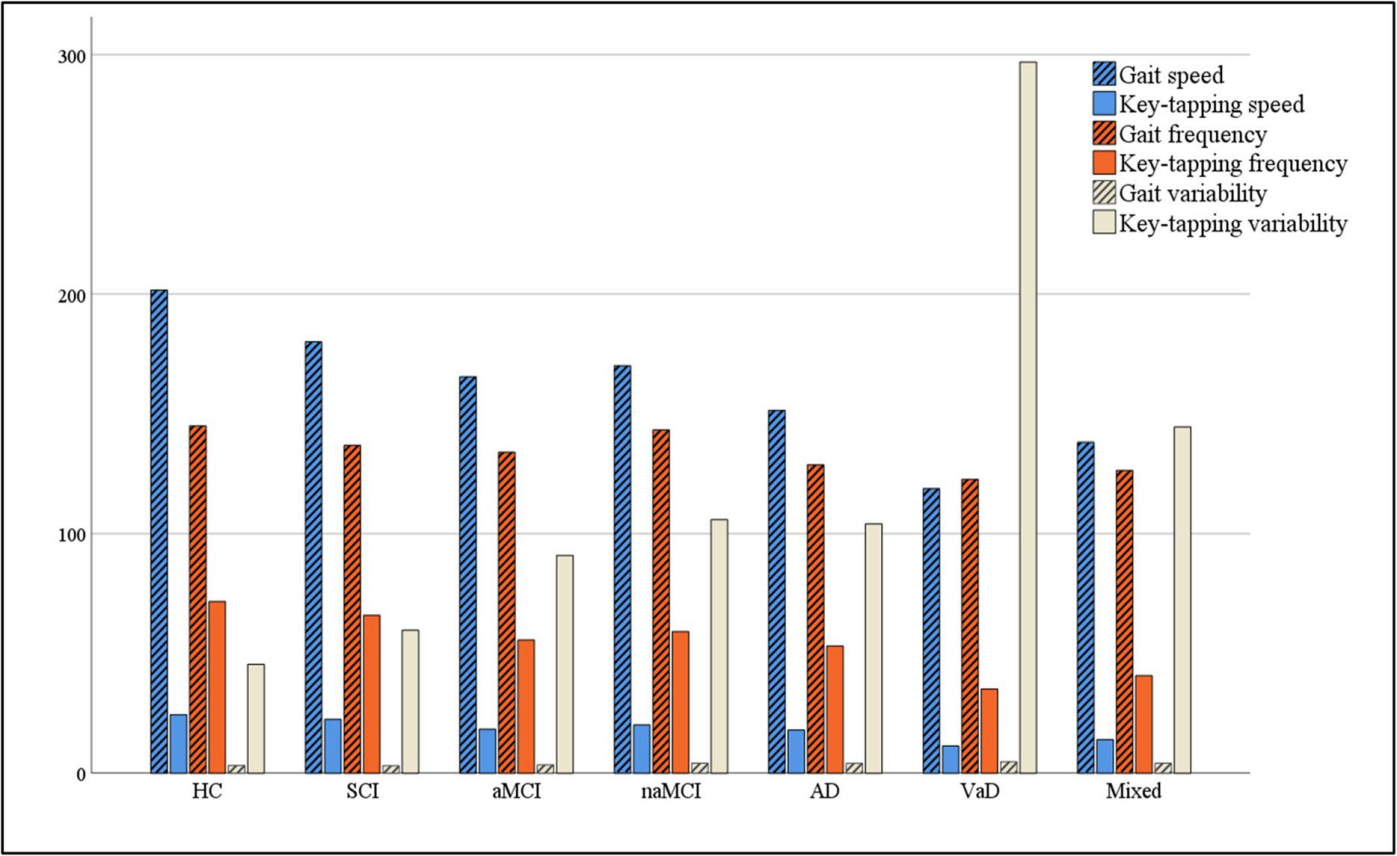
Comparison of mean of speed, frequency and variability of gait and dominant hand key-tapping between diagnostic groups/subtypes. The units are different for each variable; speed is cm/s while variability is the CV of step time for gait and key-tap time for key-tapping; stepping frequency is the number of steps/60s while key-tapping frequency is the number of key-taps/30s. HC, cognitively healthy control; SCI, subjective cognitive impairment; aMCI, amnestic mild cognitive impairment; nMCI, non-amnestic mild cognitive impairment; AD, Alzheimer’s disease; VaD, vascular dementia; Mixed, mixed AD/VaD

Participants’ characteristics are summarised in Table 1. Those with dementia were older than other groups (*P* < 0.001) and, compared to SCI and HC, had less education (*P* < 0.001). Compared to MCI and dementia, there were more women in SCI and HC groups. There were no differences in education between MCI and dementia (*P:* 0.08) or between SCI and HC (*P:* 0.92). There were no significant group differences in height, BMI and mood scores. Motor characteristics of individual groups are in Supplementary Table 2. Supplementary Tables 3 and 4 present the demographics and motor characteristics of MCI and dementia subtypes. There were no differences between subtypes of dementia, or MCI, in age, education, height, BMI and mood scores. Compared to those with AD, people with VaD had a significantly slower gait (*P*: 0.023) and key-tapping speed (*P* < 0.001) and lower key-tapping frequency (*P*: 0.001). Key-tapping variability of VaD was significantly greater than AD (*P*: 0.001) and mixed dementia (*P*: 0.006). Figure 1 presents the mean of gait and key-tapping speed, frequency and variability for all groups/subtypes.

**Table 1:** Characteristics of participants in each diagnostic group

		Dementia	MCI	SCI	HC
		*n*: 73	*n*: 106	*n*: 57	*n*: 83
Age (years)	Mean (SD)	75.60 (7.77)	70.29 (9.43)	65.14 (8.50)	64.96 (7.03)
Sex (female)	*n* (%)	39 (53)	65 (61)	39 (68)	68 (82)
Years of education	Mean (SD)	11.90 (2.87)	13.00 (3.31)	14.75 (3.23)	15.15 (2.52)
Height (m)		1.66 (.10)	1.66 (.10)	1.66 (.09)	1.63 (.08)
BMI		26.12 (4.03)	27.75 (6.39)	28.06 (5.53)	27.63 (5.07)
Right-handed	*n* (%)	69 (95)	94 (89)	53 (93)	79 (95)
Dementia subtypes					
AD	*n* (%)	43 (59)			
VaD	*n* (%)	8 (11)			
Mixed dementia	*n* (%)	22 (30)			
MCI subtypes					
aMCI	*n* (%)		68 (64)		
nMCI	*n* (%)		38 (36)		
ACE-III Scores		*n*: 68	*n*: 92	*n*: 46	*n*: 83
Total (/100)	Mean (SD)	77.75 (11.00)	88.75 (7.55)	93.91 (5.23)	97.66 (1.60)
Attention (/18)	Mean (SD)	14.71 (3.01)	16.48 (1.75)	17.43 (1.32)	17.72 (.59)
Memory (/26)	Mean (SD)	16.91 (4.53)	21.22 (4.22)	23.04 (2.51)	25.66 (.63)
Fluency (/14)	Mean (SD)	8.40 (3.21)	11.00 (2.16)	12.38 (2.00)	12.93 (.99)
Language (/26)	Mean (SD)	23.94 (2.75)	25.15 (1.36)	25.57 (.91)	25.69 (.64)
Visuo-spatial (/13)	Mean (SD)	13.79 (2.25)	14.90 (1.42)	15.74 (1.83)	15.66 (.74)
TMT—A		*n*: 55	*n*: 100	*n*: 54	*n*: 83
Time (seconds)	Mean (SD)	47.03 (19.27)	38.66 (16.78)	30.89 (10.11)	29.76 (8.11)
TMT—B		*n*: 48	*n*: 94	*n*: 54	*n*: 83
Time (seconds)	Mean (SD)	154.30 (80.70)	116.16 (62.83)	73.39 (31.58)	58.18 (15.40)
Mood Score (/15)		*n*: 72	*n*: 106	*n*: 57	*n*: 80
	Mean (SD)	4.78 (3.50)	4.84 (3.21)	4.51 (3.01)	4.79 (3.29)

*MCI* mild cognitive impairment, *SCI* subjective cognitive impairment, *HC* cognitively healthy control group, *n* number, *SD* standard deviation, *BMI* Body Mass Index, *AD* Alzheimer’s disease, *VaD* vascular dementia, *ACE-III* Addenbrooke’s Cognitive Examination-version 3 – Australian version, *TMT* Trail Making Test. Mood scores are derived from the Geriatric Depression Scale (GDS) (out of 15) and the Hospital Anxiety and Depression Scale (HADS) (out of 21) for the Clinic and healthy controls, respectively. Algebraic rearrangement was used to transform the proportion of HADS scores before analysing the differences between groups. HADS Mean (SD) before transformation of proportion was 6.70 (4.61)/21

### Association of motor variables with cognitive diagnoses (Table 2)

**Table 2:** Associations between motor variables (gait and key-tapping of dominant hand) in a model adjusted for age, sex and education

	**HC v dementia**			**HC v MCI**			**HC v SCI**		
	*β*	95% CI	*P*	*β*	95% CI	*P*	*β*	95% CI	*P*
Gait									
Speed	− 42.98	− 53.99; − 31.98	**<.001**	− 35.87	− 45.10; − 26.64	**<.001**	− 18.59	− 28.46; − 8.28	**<.001**
Frequency	− 12.77	− 18.33; − 7.21	**<.001**	− 10.05	− 14.71; − 5.38	**<.001**	− 5.21	− 10.30; −.11	.045
Variability	.76	.2; 1.31	**.006**	.78	.32; 1.23	**.001**	−.12	−.62;.38	.631
Contact	1.70	.92; 2.49	**<.001**	1.79	1.13; 2.45	**<.001**	1.02	.30; 1.74	**.006**
Key-tapping									
Speed	− 6.63	− 8.34; − 4.93	**<.001**	− 4.86	− 6.35; − 3.38	**<.001**	− 1.42	− 2.95;.11	.068
Frequency	− 18.98	− 23.88; − 14.09	**<.001**	− 12.97	− 17.26; − 8.69	**<.001**	− 4.54	− 8.94; −.15	**.043**
Variability	67.38	33.42; 101.35	**<.001**	34.95	5.34; 64.57	**.021**	9.53	− 20.97; 40.03	.538
Contact	22.91	8.78; 37.05	**.002**	7.81	− 4.51; 20.12	.213	7.34	− 5.33; 20.01	.255
	**SCI v dementia**			**SCI v MCI**			**MCI v dementia**		
	*β*	95% CI	*P*	*β*	95% CI	*P*	*β*	95% CI	*P*
Gait									
Speed	− 24.61	− 36.19; − 13.03	**<.001**	− 17.50	− 27.50; − 7.50	**.001**	− 7.11	− 16.40; 2.18	.133
Frequency	− 7.56	− 13.41; − 1.72	**.011**	− 4.84	− 9.89;.21	.060	− 2.72	− 7.41; 1.97	.254
Variability	.88	.31; 1.46	**.003**	.90	.40; 1.39	**<.001**	.02	−.48;.44	.947
Contact	.69	−.14; 1.51	.103	.77	.06; 1.49	**.031**	−.09	−.75;.57	.795
Key-tapping									
Speed	− 5.21	− 6.98; − 3.43	**<.001**	− 3.44	− 5.03; − 1.85	**<.001**	− 1.77	− 3.35; −.19	**.029**
Frequency	− 14.44	− 19.54; − 9.34	**<.001**	− 8.43	− 13.02; − 3.84	**<.001**	− 6.01	− 10.58; − 1.45	**.010**
Variability	57.85	22.51; 93.19	**.001**	25.42	− 6.30; 57.14	.116	32.43	.95; 63.91	**.044**
Contact	15.58	.85; 30.30	**.038**	.47	− 12.75; 13.69	.945	15.11	1.99; 28.23	**.024**

Bold entries indicate *p*-value 0.05

*HC* cognitively healthy controls, *MCI* mild cognitive impairment, *SCI* subjective cognitive impairment, *β* beta coefficient, *CI* confidence interval, *P P*-value

#### Associations between gait variables and cognitive diagnoses

Slower gait speed, lower frequency, greater variability and longer contact were associated with all cognitive groups (all *P*s < 0.001). Compared to HC, people with dementia had the slowest walking speed and longest contacts followed by MCI and SCI. Compared to HC, people with dementia, then MCI, had the lowest stepping frequency and greatest variability but no differences were seen in SCI. Compared to SCI, people with MCI and dementia had a slower gait speed, greater variability and longer contact. There were no differences in gait measures between MCI and dementia groups.

#### Associations between key-tapping variables and cognitive diagnoses

**Fig. 2:**
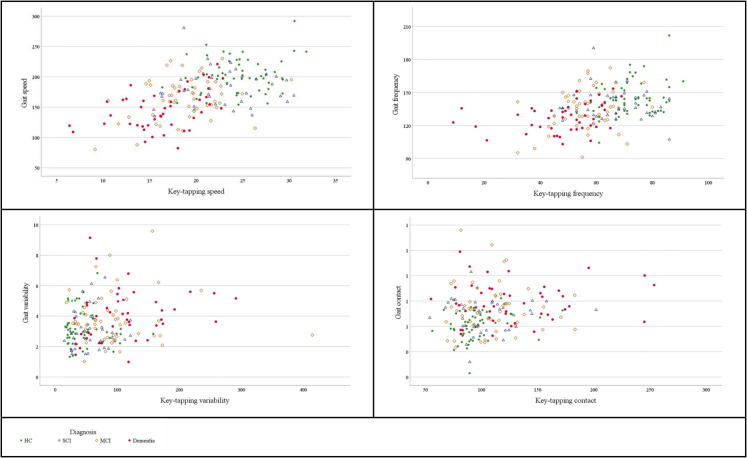
Scatterplots of the relationship between gait variables and dominant hand key-tapping variables for cognitive diagnosis groups

Slower key-tapping speed, lower frequency, greater variability and longer contact of either hand were associated with all cognitive groups (all *P*s < 0.001). Compared to HC, people with dementia had the slowest key-tapping speed and lowest frequency, followed by MCI and then SCI. Compared to HC, people with dementia, and MCI, had a significantly greater variability and longer contacts when tapping with their nondominant hand. Compared to SCI, people with dementia had a slower speed, lower frequency, greater variability and longer contact. In comparison to SCI, people with MCI had a slower key-tapping speed and lower frequency. Compared to MCI, people with dementia had a slower speed and lower tapping frequency of either hand, and greater variability when tapping with their nondominant hand. Figure 2 presents the relationship between gait variables and dominant hand key-tapping variables for cognitive diagnosis groups.

In summary, the motor associations with cognitive groups followed the same patterns of slower speed, and lower key-tapping frequency, for MCI and dementia compared to HC and SCI.

Slower key-tapping speed and lower frequency were associated with all cognitive groups, with an increase in the effect size with increase in the severity of cognitive impairment. Gait variability was associated with MCI and dementia while key-tapping variability was strongly associated with dementia, but not with MCI. Overall, stronger associations with diagnostic groups were seen for hand motor features compared to gait.

Supplementary Table 5 presents the associations between motor variables and subtypes of MCI and dementia. In summary, gait and key-tapping speed, frequency and variability were associated with AD (*P* < 0.001) and VaD (*P* ≤ 0.01). The effect size of the key-tapping variability was larger in VaD (*β*: 225.71) compared to AD (*β*: 38.30). Speed and frequency (either gait or key-tapping) were associated with both aMCI and nMCI (*P* ≤ 0.02). Gait variability (*β*: 0.88, *P*: 0.008) and key-tapping variability of either hand (*β*: 53.01, *P* ≤ 0.02) were associated with nMCI.

### Relationships between gait and key-tapping variables

#### Correlations between gait and key-tapping variables

We found moderate positive unadjusted correlations between gait speed and key-tapping speed and frequency (either hand). There was a moderate inverse correlation between gait contact and dominant hand key-tapping speed and frequency (Supplementary Table 6).

#### Associations between corresponding gait and key-tapping variables

Gait and key-tapping speed, frequency and variability were associated (all *P*s ≤ 0.03). There were no significant interactions between key-tapping variables of either hand with cognitive diagnosis. Therefore, the interaction terms were not included in the final regression model (Supplementary Table 7).

#### Classification of cognitive diagnosis groups

In Model One, only gait speed increased classification accuracy of dementia from HC (AUC: 0.94, *P*: 0.025) and MCI from HC (AUC: 0.87, *P*: 0.003) compared to the Null Model (AUCs: dementia: 0.89, MCI: 0.79) (Supplementary Table 8).

**Fig. 3:**
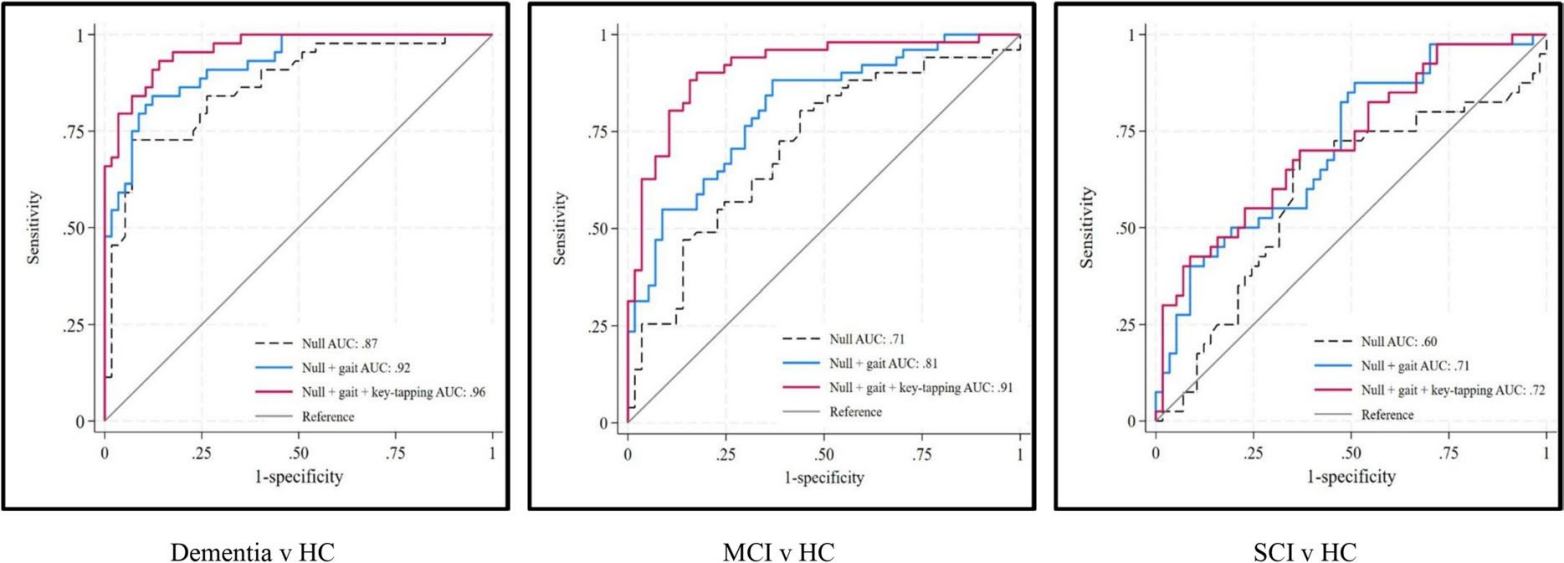
Comparison of the AUC of speed of gait and dominant hand key-tapping for discriminating cognitively healthy controls from dementia (left), from MCI (middle) and from SCI (right). HC, cognitively healthy controls; MCI, mild cognitive impairment; SCI, subjective cognitive impairment; AUC, area under the receiver-operation-characteristic curve

In Model Two, the addition of key-tapping speed and frequency of either hand improved the accuracy of classifying MCI and dementia, but not SCI, from HC compared to Model One (Table 3). Supplementary Table 9 presents Model Two results for nondominant hand. Figure 3 shows the speed AUC of the Null, Model One and Model Two for each cognitive diagnostic group.

**Table 3:** Classification accuracy of cognitive diagnoses in an adjusted model: Model Two comprises Model One (the Null Model plus a gait variable) plus the corresponding key-tapping variable of the dominant hand

	**HC v dementia**			**HC v MCI**			**HC v SCI**		
		*n*: 101			*n*: 108			*n*: 97	
	AUC	95% CI	*P*	AUC	95% CI	*P*	AUC	95% CI	*P*
Null + gait speed	.92	.87;.97		.81	.73;.89		.71	.61;.81	
Null + gait speed + key-tapping speed	.96	.93;.99	**.032**	.91	.86;.97	**.004**	.72	.62;.83	.640
Null + gait frequency	.89	.82;.95		.71	.61;.81		.62	.50;.73	
Null + gait frequency + key-tapping frequency	.96	.93;.99	**.008**	.89	.83;.95	** <.001**	.67	.57;.78	.220
Null + gait variability	.89	.83;.96		.74	.65;.84		.62	.51;.74	
Null + gait variability + key-tapping variability	.92	.87;.98	.078	.84	.77;.92	**.013**	.69	.59;.80	.249
Null + gait contact	.89	.83;.96		.75	.66;.84		.64	.53;.76	
Null + gait contact + key-tapping contact	.92	.87;.97	.141	.77	.69;.86	.227	.68	.57;.78	.293
	**SCI v dementia**			**SCI v MCI**			**MCI v dementia**		
		*n*: 84			*n*: 91			*n*: 95	
	AUC	95% CI	*P*	AUC	95% CI	*P*	AUC	95% CI	*P*
Null + gait speed	.87	.80;.95		.68	.57;.79		.71	.61;.82	
Null + gait speed + key-tapping speed	.90	.84;.97	.169	.80	.71;.90	**.015**	.74	.64;.84	.390
Null + gait frequency	.86	.78;.94		.66	.55;.77		.71	.60;.81	
Null + gait frequency + key-tapping frequency	.91	.85;.97	.054	.79	.69;.89	**.016**	.76	.66;.86	.202
Null + gait variability	.86	.78;.94		.73	.62;.84		.70	.60;.81	
Null + gait variability + key-tapping variability	.89	.82;.96	.220	.77	.68;.87	.239	.71	.61;.81	.796
Null + gait contact	.86	.77;.94		.67	.56;.78		.70	.60;.81	
Null + gait contact + key-tapping contact	.87	.79;.95	.255	.69	.58;.80	.329	.73	.61;.83	.352

Bold entries indicate *p*-value 0.05

Area under the receiver operating characteristic curve (AUC) and 95% confidence intervals for gait variables in comparison to the Null Model comprising age, sex and years of education. *P* <.05 indicates key-tapping variable improves prediction of diagnosis over and above demographic variables. Abbreviations: *HC* healthy controls, *MCI* mild cognitive impairment, *SCI* subjective cognitive impairment, *AUC* area under the receiver operating characteristic curve, *CI* confidence interval, *P* *P*-value. *ND* nondominant hand, *D* dominant hand

Combining nondominant hand key-tapping variability with the model comprising the Null Model and gait variability improved classification of dementia (AUC: 0.94, *P:* 0.029), and MCI (AUC: 0.90, *P:* 0.001), from HC. Adding key-tapping contact to the model did not aid with improving discrimination of any diagnostic group.

## Discussion

In this study, we examined the associations of upper and lower limb motor measures with cognitive diagnoses. For the first time, we evaluated the correlations between gait and key-tapping measures and evaluated classification accuracy of a combined gait and key-tapping model compared to a model with an individual motor measure or the Null Model. The main findings were that gait and key-tapping measures were associated with cognitive diagnoses across the dementia continuum, gait and key-tapping measures were moderately correlated and, combining them improved classification of MCI and dementia over and above the Null Model. These findings expand the motor-cognitive literature by examining a range of validated motor variables in larger samples, and specifically in robust, clinically-diagnosed subjective and objective cognitive groups. Our findings are a positive step towards developing clinically applicable motor biomarkers of dementia.

### Associations between motor variables and cognitive diagnoses

Slower speed, lower frequency and greater variability, of either gait or key-tapping, were associated with MCI and dementia. These findings broadly align with two previous upper and lower limb motor-cognitive studies: One study (2023) examined key-tapping of 26 people with AD, 27 aMCI and 47 HC, finding that AD, but not aMCI, had lower tapping frequency and greater variability than HC [22]. Another study (2019) measured key-tapping in 15 participants with AD, 20 preclinical AD (positive CSF Amyloid-β) and 37 HC without AD pathology [35]. They found the AD group had slower speed and greater variability compared to both preclinical and HC groups. Greater motor variability in AD and dementia is an important finding considering the blurred clinical boundaries between MCI and early-stage dementia [30, 39] and may be a valuable biomarker to aid differentiating these stages.

### Relationships between gait and key-tapping variables

Gait speed, frequency and variability were associated with conceptually equivalent key-tapping variables, but their correlations were moderate at best. Evidence from brain imaging studies may provide an explanation for this novel finding. Dementia-, especially AD-related changes in the brain are found to alter motor function networks as well as higher-order cognitive systems [40]. Increased neurodegeneration severity is associated with weakening of networks within hands’ sensory-motor systems [40, 41]. Brain imaging studies have shown that reduced hippocampal volume, a marker of AD pathology, is associated with gait slower speed and greater variability in MCI and AD [12, 42–46]. A recent study (2023), investigating key-tapping measures and brain imaging biomarkers, found greater variability was associated with reduced hippocampal volume in AD [22]. A study (2020) on association of cognitively asymptomatic brain structure with ULMF (e.g., thumb/index finger-tapping, forearm supination/pronation) also showed disrupted white matter integrity was associated with impaired ULMF but not with gait speed [47]. This, together with the weak-to-moderate correlations found between gait and key-tapping measures in our study, might suggest that although some neural motor-cognitive pathways are shared, upper and lower limb motor functions are not equivalently, or synchronously, affected by cognitive impairment and, therefore, one’s performance may not necessarily reflect the status of the other. Further exploration of these associations is important in understanding the neural pathways involved in motor-cognitive function and how they are affected in cognitive impairment.

### Classification of cognitive diagnosis groups

In the model examining gait variables, only speed classified MCI and dementia groups from HC. This is a novel finding, as to our knowledge, no study has used fast-paced gait to discriminate between cognitive groups. A longitudinal study (2019) of fast-paced gait in 223 cognitively healthy participants at baseline found faster fast-paced speed was associated with lower risk of developing dementia at 5.2 year follow-up [48]. Self-paced or dual-task gait variables have been more commonly evaluated but the results have been inconsistent. For example, a study (2022) that examined gait in 31 participants with dementia, 32 MCI and 30 HC reported gait speed (under self-paced or dual-task conditions) classified dementia from HC (AUC: 0.82) but did not discriminate MCI from HC [36]. Conversely, a study (2021) of 72 participants with AD and 214 MCI found self-paced gait speed did not discriminate AD (AUC: 0.51) or MCI (AUC: 0.58) [13].

Compared to gait, there is a paucity of studies using key-tapping tests to classify cognitive diagnosis groups, but the methods are generally more comparable as they tend to use fast-paced protocols [19, 22, 35, 38]. Our previous study found that key-tapping speed and frequency classified ≥ 94% of dementia (*n*: 50), and ≥ 88% of MCI (*n*: 58), from HC (*n*: 60) but not SCI (*n*: 40) [19]. Another recent study also reported key-tapping speed and variability classified 26 participants with AD from 47 HC (AUC: 0.78) but not aMCI (AUC: 0.60), from 47 HC [22].

In this study, we found that combined models improved discrimination of MCI and dementia from HC above the adjusted models comprising either a gait or a key-tapping variable. Only two previous studies measured a combination of gait and any ULMF; the first was a longitudinal study (2022) of 18,171 cognitively healthy participants at baseline, of whom 558 developed dementia within 4.7 years [15]. They found that a combined model of gait speed and grip strength improved prediction of conversion to all-cause dementia (91%) compared to gait speed (49%) or grip strength (28%) individually. The second study was a cross-sectional study (2021) of 26 participants with AD, 45 MCI and 47 HC, using measures of gait and drawing tasks (e.g., intersecting pentagons) [26]. Their combined model highly discriminated dementia (98%) and MCI (87%) from HC compared to models with only gait (AD: 89%, MCI: 77%) or drawing (AD: 96%, MCI: 81%) [26]. However, it is unclear what motor measures were significant in the model, and to what extent, as they trained the dataset using iterative ten-fold cross validation. In our study, in addition to a larger MCI sample and including SCI, we selected a rhythmic and repetitive hand function that is more comparable with gait than a measure of muscle strength or drawing.

Higher classification accuracy of combined models is an important finding and, together with our results on correlations between motor variables, suggest that gait and key-tapping provide information on different aspects of motor-cognitive associations and therefore presents a more comprehensive view of the changes in motor function caused by dementia pathology. An interesting finding of our study was that the nondominant hand measures were more discriminatory than the dominant hand. Another study (2023) also reported nondominant hand key-tapping variability was associated with aMCI, while both hands’ key-tapping variability was impaired in people with AD [22]. The exact mechanism behind this finding is unknown. Thus, and considering the lateralisation in the normal brain, impaired motor performance of the nondominant hand in the earlier stages of the continuum may be a feature worth further exploration [49].

### Strengths, limitations, and future directions

Our study has several strengths. We tested gait, a well-established measure of lower limb motor function, using a validated electronic gait mat in combination with a short and validated computerised upper limb test and expanded the literature by looking at fast-paced gait. We recruited relatively large samples of people with MCI and dementia and, for the first time, included a SCI group, covering all the symptomatic stages of the continuum. Additionally, we explored upper and lower limb motor features of subtypes of dementia (AD, VaD and mixed dementia) and MCI (amnestic and non-amnestic). All our participants in cognitive groups received consensus diagnosis post comprehensive interdisciplinary assessments, so the diagnosis is more robust than using a screening tool to define the groups.

The limitations are acknowledged. We did not evaluate associations with blood or brain imaging biomarkers of dementia, nor did we adjust for potential confounders such as vision or pain. We recruited participants from Tasmania where a large proportion of the older population have northern European ancestry. Compared to MCI and dementia groups, our SCI group was smaller and may be underpowered to detect associations. As a group with increased risk of developing MCI and dementia, future research could investigate larger SCI sample sizes to better understand the characteristics of this group in cross-sectional and longitudinal studies. This approach will help broaden our understanding of motor-cognitive associations and may aid developing more efficient tests to detect dementia early.

In conclusion, improved classification accuracy of MCI and dementia using a combination of gait and key-tapping variables highlights the benefits of this approach in detecting these cognitive diagnoses. The associations found between measures of gait, hand motor function and cognition warrant further investigation of these early motor biomarkers of dementia.

## Supplementary Information

Below is the link to the electronic supplementary material.

ESM 1 (DOCX 14.7 KB)

ESM 2 (DOCX 27.2 KB)

ESM 3 (DOCX 28.5 KB)

ESM 4 (DOCX 30.7 KB)

ESM 5 (DOCX 23.2 KB)

ESM 6 (DOCX 16.7 KB)

ESM 7 (DOCX 22.0 KB)

ESM 8 (DOCX 31.7 KB)

ESM 9 (DOCX 27.5 KB)

## Data Availability

The data that support the findings of this study are available on request from the corresponding author upon reasonable request.

## References

[1] Nichols E, Steinmetz JD, Vollset SE, Fukutaki K, Chalek J, Abd-Allah F, et al. Estimation of the global prevalence of dementia in 2019 and forecasted prevalence in 2050: an analysis for the Global Burden of Disease Study 2019. The Lancet Public Health. 2022;7(2):e105–25. 10.1016/S2468-2667(21)00249-8. 34998485 10.1016/S2468-2667(21)00249-8 PMC8810394

[2] Livingston G, Huntley J, Liu KY, Costafreda SG, Selbæk G, Alladi S, et al. Dementia prevention, intervention, and care: 2024 report of the Lancet standing Commission. The Lancet. 2024;404(10452):572–628. 10.1016/s0140-6736(24)01296-0. 10.1016/S0140-6736(24)01296-0 39096926

[3] Mitchell AJ, Beaumont H, Ferguson D, Yadegarfar M, Stubbs B. Risk of dementia and mild cognitive impairment in older people with subjective memory complaints: meta-analysis. Acta Psychiatr Scand. 2014;130(6):439–51. 10.1111/acps.12336. 25219393 10.1111/acps.12336

[4] Rudd KD, Lawler K, Callisaya ML, Alty J. Investigating the associations between upper limb motor function and cognitive impairment: a scoping review. GeroScience. 2023;45(6):3449–73. 10.1007/s11357-023-00844-z. 37337026 10.1007/s11357-023-00844-z PMC10643613

[5] Buchman AS, Yu L, Wilson RS, Boyle PA, Schneider JA, Bennett DA, et al. Brain pathology contributes to simultaneous change in physical frailty and cognition in old age. The Journals of Gerontology: Series A. 2014;69(12):1536–44. 10.1093/gerona/glu117. 10.1093/gerona/glu117 PMC4296120 25136002

[6] Montero-Odasso M, Speechley M, Muir-Hunter SW, Sarquis-Adamson Y, Sposato LA, Hachinski V, et al. Motor and cognitive trajectories before dementia: results from gait and brain study. J Am Geriatr Soc. 2018;66(9):1676–83. 10.1111/jgs.15341. 29608780 10.1111/jgs.15341

[7] Hausdorff JM, Buchman AS. What links gait speed and MCI with dementia? A fresh look at the association between motor and cognitive function. The Journals of Gerontology: Series A. 2013;68(4):409–11. 10.1093/gerona/glt002. 10.1093/gerona/glt002 PMC3593618 23401565

[8] Basile G, Sardella A. From cognitive to motor impairment and from sarcopenia to cognitive impairment: a bidirectional pathway towards frailty and disability. Aging Clin Exp Res. 2021;33(2):469–78. 10.1007/s40520-020-01550-y. 32277434 10.1007/s40520-020-01550-y

[9] Grande G, Triolo F, Nuara A, Welmer A-K, Fratiglioni L, Vetrano DL. Measuring gait speed to better identify prodromal dementia. Exp Gerontol. 2019;124: 110625. 10.1016/j.exger.2019.05.014. 31173841 10.1016/j.exger.2019.05.014

[10] Abellan Van Kan G, Rolland Y, Andrieu S, Bauer J, Beauchet O, Bonnefoy M, et al. Gait speed at usual pace as a predictor of adverse outcomes in community-dwelling older people an International Academy on Nutrition and Aging (IANA) Task Force. The Journal of nutrition health and aging. 2009;13(10):881–9. 10.1007/s12603-009-0246-z. 10.1007/s12603-009-0246-z PMC12878092 19924348

[11] Veronese N, Stubbs B, Trevisan C, Bolzetta F, De Rui M, Solmi M, et al. What physical performance measures predict incident cognitive decline among intact older adults? A 4.4 year follow up study. Experimental Gerontology. 2016;81:110–8. 10.1016/j.exger.2016.05.008. 27235850 10.1016/j.exger.2016.05.008

[12] Rosso AL, Verghese J, Metti AL, Boudreau RM, Aizenstein HJ, Kritchevsky S, et al. Slowing gait and risk for cognitive impairment: the hippocampus as a shared neural substrate. Neurology. 2017;89(4):336–42. 10.1212/WNL.0000000000004153. 28659421 10.1212/WNL.0000000000004153 PMC5574674

[13] Pieruccini-Faria F, Black SE, Masellis M, Smith EE, Almeida QJ, Li KZH, et al. Gait variability across neurodegenerative and cognitive disorders: results from the Canadian Consortium of Neurodegeneration in Aging (CCNA) and the Gait and Brain Study. Alzheimers Dement. 2021;17(8):1317–28. 10.1002/alz.12298. 33590967 10.1002/alz.12298 PMC8451764

[14] Montero-Odasso MM, Sarquis-Adamson Y, Speechley M, Borrie MJ, Hachinski VC, Wells J, et al. Association of dual-task gait with incident dementia in mild cognitive impairment: results from the gait and brain study. JAMA Neurol. 2017;74(7):857–65. 10.1001/jamaneurol.2017.0643. 28505243 10.1001/jamaneurol.2017.0643 PMC5710533

[15] Orchard SG, Polekhina G, Ryan J, Shah RC, Storey E, Chong TT-J, et al. Combination of gait speed and grip strength to predict cognitive decline and dementia. Alzheimer’s & Dementia: Diagnosis, Assessment & Disease Monitoring. 2022;14(1):e12353. 10.1002/dad2.12353. 10.1002/dad2.12353 PMC9494608 36187193

[16] Tajimi T, Furuta Y, Hirabayashi N, Honda T, Hata J, Ohara T, et al. Association of gait speed with regional brain volumes and risk of dementia in older Japanese: The Hisayama study. Arch Gerontol Geriatr. 2023;106: 104883. 10.1016/j.archger.2022.104883. 36495658 10.1016/j.archger.2022.104883

[17] Tian Q, Chastan N, Bair W-N, Resnick SM, Ferrucci L, Studenski SA. The brain map of gait variability in aging, cognitive impairment and dementia—a systematic review. Neurosci Biobehav Rev. 2017;74:149–62. 10.1016/j.neubiorev.2017.01.020. 28115194 10.1016/j.neubiorev.2017.01.020 PMC5303129

[18] Montero-Odasso M, Pieruccini-Faria F, Ismail Z, Li K, Lim A, Phillips N, et al. CCCDTD5 recommendations on early non cognitive markers of dementia: a Canadian consensus. Alzheimer’s & Dementia: Translational Research & Clinical Interventions. 2020;6(1): e12068. 10.1002/trc2.12068. 33094146 10.1002/trc2.12068 PMC7568425

[19] Rudd, KD, Lawler, K, Callisaya, ML, Bindoff, AD, Chiranakorn-Costa, S, Li, R, et al. (2024). Hand motor dysfunction is associated with both subjective and objective cognitive impairment across the dementia continuum. Dementia and Geriatric Cognitive Disorders. 1-. 10.1159/000540412 10.1159/000540412 39074458

[20] Kobayashi-Cuya KE, Sakurai R, Sakuma N, Suzuki H, Ogawa S, Takebayashi T, et al. Bidirectional associations of high-level cognitive domains with hand motor function and gait speed in high-functioning older adults: a 7-year study. Arch Gerontol Geriatr. 2024;117: 105232. 10.1016/j.archger.2023.105232. 37956584 10.1016/j.archger.2023.105232

[21] Wang X, St George RJ, Bindoff AD, Noyce AJ, Lawler K, Roccati E, et al. Estimating presymptomatic episodic memory impairment using simple hand movement tests: a cross-sectional study of a large sample of older adults. Alzheimers Dement. 2024;20(1):173–82. 10.1002/alz.13401. 37519032 10.1002/alz.13401 PMC10916999

[22] Koppelmans V, Ruitenberg MFL, Schaefer SY, King JB, Hoffman JM, Mejia AF, et al. Delayed and more variable unimanual and bimanual finger tapping in Alzheimer’s disease: associations with biomarkers and applications for classification. J Alzheimers Dis. 2023;95:1233–52. 10.3233/jad-221297. 37694362 10.3233/JAD-221297 PMC10578230

[23] Callisaya ML, Blizzard CL, Wood AG, Thrift AG, Wardill T, Srikanth VK. Longitudinal relationships between cognitive decline and gait slowing: the Tasmanian Study of Cognition and Gait. The Journals of Gerontology: Series A. 2015;70(10):1226–32. 10.1093/gerona/glv066. 10.1093/gerona/glv066 26009641

[24] Cui M, Zhang S, Liu Y, Gang X, Wang G. Grip strength and the risk of cognitive decline and dementia: a systematic review and meta-analysis of longitudinal cohort studies. Frontiers in aging neuroscience. 2021;13: 625551. 10.3389/fnagi.2021.625551. 33613270 10.3389/fnagi.2021.625551 PMC7890203

[25] Kumar S, Rani S, Sharma S, Min H. Multimodality fusion aspects of medical diagnosis: a comprehensive review. Bioengineering. 2024;11(12):1233. 10.3390/bioengineering11121233. 39768051 10.3390/bioengineering11121233 PMC11672922

[26] Yamada Y, Shinkawa K, Kobayashi M, Caggiano V, Nemoto M, Nemoto K, et al. Combining multimodal behavioral data of gait, speech, and drawing for classification of Alzheimer’s disease and mild cognitive impairment. Journal of Alzheimer’s Disease. 2021;84:315–27. 10.3233/JAD-210684. 34542076 10.3233/JAD-210684 PMC8609704

[27] Borda MG, Reyes-Ortiz C, Pérez-Zepeda MU, Patino-Hernandez D, Gómez-Arteaga C, Cano-Gutiérrez CA. Educational level and its association with the domains of the Montreal Cognitive Assessment Test. Aging Ment Health. 2019;23(10):1300–6. 10.1080/13607863.2018.1488940. 30449144 10.1080/13607863.2018.1488940

[28] Alty J, Lawler K, Salmon K, McDonald S, Stuart K, Cleary A, et al. A new one-stop interdisciplinary cognitive clinic model tackles rural health inequality and halves the time to diagnosis: benchmarked against a national dementia registry. Int J Geriatr Psychiatry. 2023;38(8): e5988. 10.1002/gps.5988. 37592719 10.1002/gps.5988

[29] Sachdev PS, Blacker D, Blazer DG, Ganguli M, Jeste DV, Paulsen JS, et al. Classifying neurocognitive disorders: the DSM-5 approach. Nat Rev Neurol. 2014;10(11):634–42. 10.1038/nrneurol.2014.181. 25266297 10.1038/nrneurol.2014.181

[30] Petersen RC. Mild cognitive impairment. Continuum (Minneapolis, Minn). 2016;22(2):404–18. 10.1212/CON.0000000000000313. 27042901 10.1212/CON.0000000000000313 PMC5390929

[31] Bartlett L, Doherty K, Farrow M, Kim S, Hill E, King A, et al. Island study linking aging and neurodegenerative disease (ISLAND) targeting dementia risk reduction: protocol for a prospective web-based cohort study. JMIR Research Protocols. 2022;11(3): e34688. 10.2196/34688. 35230251 10.2196/34688 PMC8924774

[32] Fitzpatrick AL, Kuller LH, Lopez OL, Diehr P, O’Meara ES, Longstreth WT Jr, et al. Midlife and late-life obesity and the risk of dementia: cardiovascular health study. Arch Neurol. 2009;66(3):336–42. 10.1001/archneurol.2008.582. 19273752 10.1001/archneurol.2008.582 PMC3513375

[33] Beauchet O, Allali G, Sekhon H, Verghese J, Guilain S, Steinmetz J-P, et al. Guidelines for assessment of gait and reference values for spatiotemporal gait parameters in older adults: the biomathics and Canadian gait consortiums initiative. Front Hum Neurosci. 2017;11:353. 10.3389/fnhum.2017.00353. 28824393 10.3389/fnhum.2017.00353 PMC5540886

[34] Noyce AJ, Nagy A, Acharya S, Hadavi S, Bestwick JP, Fearnley J, et al. Bradykinesia-akinesia incoordination test: validating an online keyboard test of upper limb function. PLoS ONE. 2014;9(4): e96260. 10.1371/journal.pone.0096260. 24781810 10.1371/journal.pone.0096260 PMC4004565

[35] Mollica MA, Tort-Merino A, Navarra J, Fernández-Prieto I, Valech N, Olives J, et al. Early detection of subtle motor dysfunction in cognitively normal subjects with amyloid-β positivity. Cortex. 2019;121:117–24. 10.1016/j.cortex.2019.07.021. 31561128 10.1016/j.cortex.2019.07.021

[36] Bovonsunthonchai S, Vachalathiti R, Hiengkaew V, Bryant MS, Richards J, Senanarong V. Quantitative gait analysis in mild cognitive impairment, dementia, and cognitively intact individuals: a cross-sectional case-control study. BMC Geriatr. 2022;22(1):767. 10.1186/s12877-022-03405-9. 36151524 10.1186/s12877-022-03405-9 PMC9502583

[37] Maldonado G, Greenland S. Simulation study of confounder-selection strategies. Am J Epidemiol. 1993;138(11):923–36. 10.1093/oxfordjournals.aje.a116813. 8256780 10.1093/oxfordjournals.aje.a116813

[38] Koppelmans, V, Ruitenberg, MFL, Schaefer, SY, King, JB, Jacobo, JM, Silvester, BP, et al. Classification of mild cognitive impairment and Alzheimer’s disease using manual motor measures. Neurodegenerative Diseases. 2024:54–70. 10.1159/000539800 10.1159/000539800 PMC11381162 38865972

[39] Sabbagh MN, Boada M, Borson S, Chilukuri M, Dubois B, Ingram J, et al. Early detection of mild cognitive impairment (MCI) in primary care. The Journal of prevention of Alzheimer’s disease. 2020;7:165–70. 10.14283/jpad.2020.21. 32463069 10.14283/jpad.2020.21

[40] Zhang Z, Chan MY, Han L, Carreno CA, Winter-Nelson E, Wig GS, et al. Dissociable effects of Alzheimer’s disease-related cognitive dysfunction and aging on functional brain network segregation. J Neurosci. 2023;43(46):7879–92. 10.1523/JNEUROSCI.0579-23.2023. 37714710 10.1523/JNEUROSCI.0579-23.2023 PMC10648516

[41] Manelis A, Hu H, Satz S. The relationship between reduced hand dexterity and brain structure abnormality in older adults. Geriatrics. 2024;9(6):165. 10.3390/geriatrics9060165. 39727824 10.3390/geriatrics9060165 PMC11728121

[42] Park HY, Suh CH, Heo H, Shim WH, Kim SJ. Diagnostic performance of hippocampal volumetry in Alzheimer’s disease or mild cognitive impairment: a meta-analysis. Eur Radiol. 2022;32(10):6979–91. 10.1007/s00330-022-08838-9. 35507052 10.1007/s00330-022-08838-9

[43] Rao G, Gao H, Wang X, Zhang J, Ye M, Rao L. MRI measurements of brain hippocampus volume in relation to mild cognitive impairment and Alzheimer disease: a systematic review and meta-analysis. Medicine. 2023;102(36): e34997. 10.1097/md.0000000000034997. 37682140 10.1097/MD.0000000000034997 PMC10489245

[44] Arrondo P, Elía-Zudaire Ó, Martí-Andrés G, Fernández-Seara MA, Riverol M. Grey matter changes on brain MRI in subjective cognitive decline: a systematic review. Alzheimer’s Research & Therapy. 2022;14(1):98. 10.1186/s13195-022-01031-6. 10.1186/s13195-022-01031-6 PMC9306106 35869559

[45] Hu H-Y, Ou Y-N, Shen X-N, Qu Y, Ma Y-H, Wang Z-T, et al. White matter hyperintensities and risks of cognitive impairment and dementia: a systematic review and meta-analysis of 36 prospective studies. Neurosci Biobehav Rev. 2021;120:16–27. 10.1016/j.neubiorev.2020.11.007. 33188821 10.1016/j.neubiorev.2020.11.007

[46] Beauchet O, Launay CP, Sekhon H, Montembeault M, Allali G. Association of hippocampal volume with gait variability in pre-dementia and dementia stages of Alzheimer disease: results from a cross-sectional study. Exp Gerontol. 2019;115:55–61. 10.1016/j.exger.2018.11.010. 30447261 10.1016/j.exger.2018.11.010

[47] Zhai F, Liu J, Su N, Han F, Zhou L, Ni J, et al. Disrupted white matter integrity and network connectivity are related to poor motor performance. Sci Rep. 2020;10(1):18369. 10.1038/s41598-020-75617-1. 33110225 10.1038/s41598-020-75617-1 PMC7591496

[48] Rosso AL, Metti AL, Faulkner K, Redfern M, Yaffe K, Launer L, et al. Complex walking tasks and risk for cognitive decline in high functioning older adults. Journal of Alzheimer’s Disease. 2019;71(s1):S65–73. 10.3233/JAD-181140. 30814353 10.3233/JAD-181140 PMC6703970

[49] Toga AW, Thompson PM. Mapping brain asymmetry. Nat Rev Neurosci. 2003;4(1):37–48. 10.1038/nrn1009. 12511860 10.1038/nrn1009

