# Supplementary material for: Stepping and tapping: combining motor tasks improves cognitive classification
Source: GeroScience. 2025 May 8;48(1):829–42. doi: 10.1007/s11357-025-01678-7 (PMC12972407; doi:10.1007/s11357-025-01678-7)
Supplement: Supplementary file 1 — (DOCX 14.7 KB) [file 11357_2025_1678_MOESM1_ESM.docx]

**Supplementary Table 1.** Definitions of gait and key-tapping measures

|  | Gait | Key-tapping |
| --- | --- | --- |
| Speed | Mean walking speed | Mean inter-tap speed |
| Frequency | Number of steps per minute | Number of key-taps per 30 seconds |
| Variability | Coefficient of variation of mean step time | Coefficient of variation of mean key-tap time |
| Contact | Mean time (seconds) with a foot in contact with the gait mat | Mean time (milliseconds) spent with a fingertip depressing a key |

Measures of gait and key-tapping are conceptually comparable.
