# Supplementary material for: Stepping and tapping: combining motor tasks improves cognitive classification
Source: GeroScience. 2025 May 8;48(1):829–42. doi: 10.1007/s11357-025-01678-7 (PMC12972407; doi:10.1007/s11357-025-01678-7)
Supplement: Supplementary file 2 — (DOCX 27.2 KB) [file 11357_2025_1678_MOESM2_ESM.docx]

**Supplementary Table 2.** Gait and key-tapping characteristics of each diagnostic group.

|  |  | **Dementia** | **MCI** | **SCI** | **HC** |
| --- | --- | --- | --- | --- | --- |
| **Key-tapping** | n | 45 | 51 | 40 | 57 |
| Speed (N) | mean (SD) | 15.34 (4.18) | 18.36 (3.34) | 21.34 (3.57) | 23.46 (3.45) |
| Speed (D) | mean (SD) | 16.23 (4.17) | 18.74 (3.84) | 22.79 (4.21) | 24.44 (3.45) |
| Frequency (N) | mean (SD) | 43.02 (12.32) | 50.98 (10.15) | 59.75 (10.94) | 65.88 (8.00) |
| Frequency (D) | mean (SD) | 47.70 (14.05) | 56.32 (9.82) | 66.73 (11.00) | 71.74 (9.19) |
| Variability (N) | mean (SD) | 142.01 (129.60) | 86.68 (35.47) | 66.44 (40.61) | 45.80 (19.84) |
| Variability (D) | mean (SD) | 139.22 (136.38) | 93.59 (64.02) | 58.63 (27.11) | 45.47 (29.20) |
| Contact (N) | mean (SD) | 170.69 (55.87) | 145.71 (36.43) | 133.86 (47.75) | 116.18 (23.54) |
| Contact (D) | mean (SD) | 130.89 (48.44) | 108.93 (27.71) | 103.87 (28.28) | 95.78 (20.33) |
|  |  |  |  |  |  |
| **Gait** | n | 71 | 105 | 57 | 83 |
| Speed | mean (SD) | 142.38 (33.28) | 156.70 (33.19) | 181.55 (29.08) | 199.07 (30.43) |
| Frequency | mean (SD) | 125.16 (14.07) | 130.56 (17.22) | 138.16 (15.68) | 144.88 (15.39) |
| Variability | mean (SD) | 4.17 (1.63) | 4.11 (1.68) | 3.15 (1.19) | 3.30 (1.17) |
| Contact | mean (SD) | .61 (.09) | .59 (.09) | .54 (.07) | .51 (.06) |

Abbreviations: MCI, mild cognitive impairment; SCI, subjective cognitive impairment; HC, healthy control group; n, number; N, nondominant hand; D, dominant hand; SD, standard deviation.
