# Supplementary material for: Stepping and tapping: combining motor tasks improves cognitive classification
Source: GeroScience. 2025 May 8;48(1):829–42. doi: 10.1007/s11357-025-01678-7 (PMC12972407; doi:10.1007/s11357-025-01678-7)
Supplement: Supplementary file 3 — (DOCX 28.5 KB) [file 11357_2025_1678_MOESM3_ESM.docx]

**Supplementary Table 3.** Demographic characteristics of dementia and MCI subtypes.

|  |  | AD | VaD | Mixed | aMCI | nMCI |
| --- | --- | --- | --- | --- | --- | --- |
|  |  | n: 43 | n: 8 | n: 22 | n: 68 | n: 38 |
| Age (years) | mean (SD) | 73.88 (9.17) | 78.75 (3.88) | 77.82 (4.35) | 70.47 (8.65) | 69.97 (10.81) |
| Sex (Female) | n (%) | 25 (58) | 3 (38) | 11 (50) | 35 (52) | 30 (79) |
| Yeas of Education | mean (SD) | 12.43 (2.86) | 11.86 (4.12) | 10.91 (2.18) | 12.87 (3.09) | 13.24 (3.70) |
| Height (m) |  | 1.66 (.10) | 1.65 (.10) | 1.65 (.11) | 1.66 (.11) | 1.64 (.09) |
| BMI |  | 26.11 (4.24) | 24.80 (3.41) | 26.62 (3.88) | 27.98 (6.70) | 27.33 (5.86) |
| Right-handed | n (%) | 41 (95) | 7 (88) | 21 (96) | 66 (91) | 32 (84) |
| ACE-III Scores |  | n: 42 | n: 7 | n: 19 | n: 56 | n: 36 |
| Total (/100) | mean (SD) | 78.91 (10.34) | 79.86 (11.13) | 74.42 (12.19) | 87.46 (8.48) | 90.75 (5.33) |
| Attention (/18) | mean (SD) | 14.95 (2.85) | 14.71 (4.00) | 14.16 (3.06) | 16.38 (1.80) | 16.64 (1.69) |
| Memory (/26) | mean (SD) | 16.91 (4.64) | 19.43 (4.24) | 16.00 (4.23) | 20.11 (4.57) | 22.94 (2.92) |
| Fluency (/14) | mean (SD) | 8.98 (3.29) | 8.14 (2.55) | 7.21 (3.03) | 11.00 (2.35) | 10.89 (1.86) |
| Language (/26) | mean (SD) | 24.17 (1.81) | 24.14 (2.55) | 23.37 (2.59) | 25.07 (1.54) | 25.28 (1.03) |
| Visuo-spatial (/13) | mean (SD) | 13.91 (2.24) | 13.43 (3.05) | 13.68 (2.06) | 14.84 (1.39) | 15.00 (1.49) |
| TMT - A |  | n: 34 | n: 5 | n: 16 | n: 63 | n: 37 |
| Time (s) | mean (SD) | 42.77 (15.38) | 51.86 (19.78) | 54.56 (24.57) | 40.40 (19.07) | 35.71 (11.55) |
| TMT - B |  | n: 32 | n: 4 | n: 12 | n: 60 | n: 34 |
| Time (s) | mean (SD) | 134.17 (64.70) | 251.64 (115.74) | 174.50 (85.20) | 122.53 (67.06) | 104.91 (53.69) |
| Mood Score |  | n: 43 | n: 7 | n: 22 | n: 68 | n: 38 |
| (/15) |  | 4.63 (3.92) | 4.14 (2.34) | 5.27 (2.88) | 4.68 (3.17) | 5.13 (3.31) |

Abbreviations: AD, Alzheimer’s Disease; VaD, Vascular dementia; Mixed, mixed Alzheimer’s disease and vascular dementia; aMCI, amnestic mild cognitive impairment; nMCI, non-amnestic mild cognitive impairment; n, number; SD, standard deviation; BMI, Body Mass Index; ACE-III, Addenbrooke’s Cognitive Examination-version 3 – Australian version; TMT, Trail Making Test. Mood scores are derived from Geriatric Depression Scale (GDS) and the Hospital Anxiety and Depression Scale (HADS) for the Clinic and healthy controls respectively.
