# Supplementary material for: Stepping and tapping: combining motor tasks improves cognitive classification
Source: GeroScience. 2025 May 8;48(1):829–42. doi: 10.1007/s11357-025-01678-7 (PMC12972407; doi:10.1007/s11357-025-01678-7)
Supplement: Supplementary file 4 — (DOCX 30.7 KB) [file 11357_2025_1678_MOESM4_ESM.docx]

**Supplementary Table 4.** Gait and key-tapping characteristics of dementia and MCI subtypes.

|  |  | **AD** | **VaD** | **Mixed** | **aMCI** | **nMCI** |
| --- | --- | --- | --- | --- | --- | --- |
| **Key-tapping** | n | 30 | 6 | 9 | 35 | 16 |
| Speed (N) | mean (SD) | 17.02 (3.20) | 10.34 (4.23) | 13.11 (3.57) | 18.12 (3.00) | 18.87 (4.10) |
| Speed (D) | mean (SD) | 18.05 (3.00) | 11.39 (4.11) | 13.40 (3.67) | 18.30 (3.33) | 19.71 (4.75) |
| Frequency (N) | mean (SD) | 47.2 (11.31) | 30.83 (10.74) | 37.22 (9.18) | 50.2 (9.71) | 52.70 (11.18) |
| Frequency (D) | mean (SD) | 52.77 (11.06) | 35.17 (15.80) | 39.11 (13.74) | 55.15 (8.50) | 58.81 (12.08) |
| Variability (N) | mean (SD) | 117.02 (69.53) | 298.70 (279.11) | 120.82 (69.92) | 86.00 (32.77) | 88.23 (41.90) |
| Variability (D) | mean (SD) | 105.46 (62.55) | 296.91 (310.69) | 146.66 (69.26) | 91.58 (65.90) | 98.00 (61.55) |
| Contact (N) | mean (SD) | 151.60 (8.24) | 196.21 (22.81) | 217.34 (19.79) | 147.31 (6.49) | 142.21 (8.17) |
| Contact (D) | mean (SD) | 119.21 (7.08) | 146.55 (27.23) | 159.39 (18.47) | 109.80 (4.88) | 107.04 (6.46) |
|  |  |  |  |  |  |  |
| **Gait** | n | 43 | 8 | 20 | 67 | 38 |
| Speed | mean (SD) | 149.81 (36.91) | 122.34 (15.65) | 134.44 (24.85) | 158.53 (34.87) | 153.47 (30.19) |
| Frequency | mean (SD) | 127.49 (14.21) | 119.90 (16.45) | 122.26 (12.26) | 130.38 (19.03) | 130.88 (13.70) |
| Variability | mean (SD) | 4.25 (1.88) | 4.67 (.90) | 3.80 (1.20) | 4.00 (1.57) | 4.33 (1.90) |
| Contact | mean (SD) | 62.42 (2.48) | 61.88 (2.08) | 63.00 (1.40) | 62.38 (2.46) | 62.22 (2.33) |

Abbreviations: AD, Alzheimer’s Disease; VaD, Vascular dementia; Mixed, mixed Alzheimer’s disease and vascular dementia; aMCI, amnestic mild cognitive impairment; nMCI, non-amnestic mild cognitive impairment; n, number; N, nondominant hand; D, dominant hand; SD, standard deviation.
