# Supplementary material for: Stepping and tapping: combining motor tasks improves cognitive classification
Source: GeroScience. 2025 May 8;48(1):829–42. doi: 10.1007/s11357-025-01678-7 (PMC12972407; doi:10.1007/s11357-025-01678-7)
Supplement: Supplementary file 5 — (DOCX 23.2 KB) [file 11357_2025_1678_MOESM5_ESM.docx]

**Supplementary Table 5.** Associations between gait and key-tapping variables and subtypes of mild cognitive impairment and dementia in a model adjusted for age, sex and education.

|  | **AD** | | | **VaD** | | | **Mixed dementia** | | | **aMCI** | | | **nMCI** | | |
| --- | --- | --- | --- | --- | --- | --- | --- | --- | --- | --- | --- | --- | --- | --- | --- |
|  | β | 95% CI | *P* | β | 95% CI | *P* | β | 95% CI | *P* | β | 95% CI | *P* | β | 95% CI | *P* |
| **Gait** |  |  |  |  |  |  |  |  |  |  |  |  |  |  |  |
| speed | -40.60 | -51.70; -29.51 | <.001 | -62.89 | -85.53; -40.26 | <.001 | -49.80 | -65.82; -33.78 | <.001 | -34.52 | -44.96; -24.09 | <.001 | -33.12 | -46.13; -20.10 | <.001 |
| Frequency | -12.09 | -17.74; -6.44 | <.001 | -16.11 | -27.63; -4.59 | .006 | -14.84 | -23.00; -6.68 | <.001 | -9.64 | -14.95; -4.33 | <.001 | -8.24 | -14.87; -1.62 | .015 |
| Variability | 1.02 | .47; 1.57 | <.001 | 1.22 | .10; 2.34 | .033 | .34 | -.45; 1.14 | .395 | .52 | .01; 1.04 | .048 | .88 | .23; 1.52 | .008 |
| Contact | .07 | .04; .10 | <.001 | .08 | .02; .14 | .009 | .09 | .05; .13 | <.001 | .06 | .03; .09 | <.001 | .044 | .01; .08 | .012 |
|  |  |  |  |  |  |  |  |  |  |  |  |  |  |  |  |
|  | β | 95% CI | *P* | β | 95% CI | *P* | β | 95% CI | *P* | β | 95% CI | *P* | β | 95% CI | *P* |
| **Key-tapping** |  |  |  |  |  |  |  |  |  |  |  |  |  |  |  |
| Speed (N) | -5.36 | -6.97; -3.76 | <.001 | -11.70 | -14.71; -8.68 | <.001 | -8.72 | -11.35; -6.10 | <.001 | -4.32 | -5.84; -2.79 | <.001 | -4.50 | -6.65; -2.34 | <.001 |
| Speed (D) | -5.11 | -6.79; -3.42 | <.001 | -11.15 | -14.31; -7.98 | <.001 | -8.84 | -11.59; -6.08 | <.001 | -4.84 | -6.44; -3.24 | <.001 | -4.24 | -6.50; -1.98 | <.001 |
| Frequency (N) | -14.00 | -18.49; -9.50 | <.001 | -28.93 | -37.35; -20.50 | <.001 | -21.72 | -29.06; -14.38 | <.001 | -12.11 | -16.38; -7.84 | <.001 | -12.66 | -18.69; -6.62 | <.001 |
| Frequency (D) | -14.21 | -18.98; -9.45 | <.001 | -30.44 | -39.39; -21.49 | <.001 | -25.28 | -33.08; -17.48 | <.001 | -12.62 | -17.19; -8.05 | <.001 | -11.86 | -18.26; -5.46 | <.001 |
| Variability (N) | 66.98 | 37.48; 96.48 | <.001 | 251.47 | 196.07; 306.88 | <.001 | 73.20 | 24.94; 121.47 | .003 | 38.43 | 10.39; 66.47 | .008 | 50.28 | 10.67; 89.88 | .013 |
| Variability (D) | 38.30 | 5.75; 70.85 | .021 | 225.71 | 164.58; 286.83 | <.001 | 66.91 | 13.67; 120.16 | .014 | 30.27 | -.67; 61.20 | .055 | 53.01 | 9.32; 96.71 | .018 |
| Contact (N) | 19.64 | 1.66; 37.63 | .032 | 61.82 | 28.00; 95.64 | <.001 | 78.78 | 49.32; 108.24 | <.001 | 23.09 | 6.00; 40.18 | .008 | 19.22 | -4.93; 43.37 | .118 |
| Contact (D) | 11.10 | -3.36; 25.55 | .132 | 40.11 | 12.93; 67.29 | .004 | 48.09 | 24.41; 71.76 | <.001 | 8.10 | -5.64; 21.83 | .246 | 10.91 | -8.50; 30.31 | .269 |

Abbreviations: AD, Alzheimer’s disease; VaD, vascular dementia; Mixed, mixed Alzheimer’s disease and vascular dementia. aMCI, amnestic mild cognitive impairment; nMCI, non-amnestic mild cognitive impairment; β, Beta Coefficient; CI, Confidence Interval; *P*, p-value; D, dominant hand; N, nondominant hand.
