# Supplementary material for: Stepping and tapping: combining motor tasks improves cognitive classification
Source: GeroScience. 2025 May 8;48(1):829–42. doi: 10.1007/s11357-025-01678-7 (PMC12972407; doi:10.1007/s11357-025-01678-7)
Supplement: Supplementary file 6 — (DOCX 16.7 KB) [file 11357_2025_1678_MOESM6_ESM.docx]

**Supplementary Table 6.** Pearson’s Correlations between conceptually equivalent variables of gait and key-tapping variables of the dominant and nondominant hand.

| **Key-tapping** | Speed | | Frequency | | Variability | | Contact | |
| --- | --- | --- | --- | --- | --- | --- | --- | --- |
|  | N | D | N | D | N | D | N | D |
| **Gait** |  |  |  |  |  |  |  |  |
| Speed | .511 | .527 | .489 | .531 | -.376 | -.381 | -.322 | -.316 |
| Frequency | .364 | .379 | .360 | .385 | -.231 | -.246 | -.172 | -.213 |
| Variability | -.244 | -.239 | -.231 | -.243 | .235 | .197 | .132 | .103 |
| Contact | -.401 | -.423 | -.381 | -.420 | .243 | .265 | .150 | .198 |

The observed Correlation Coefficient (r) is weak (.10 - .39) to moderate (.40 - .69). Abbreviations: N, nondominant hand; D, dominant hand.
