# Supplementary material for: Stepping and tapping: combining motor tasks improves cognitive classification
Source: GeroScience. 2025 May 8;48(1):829–42. doi: 10.1007/s11357-025-01678-7 (PMC12972407; doi:10.1007/s11357-025-01678-7)
Supplement: Supplementary file 7 — (DOCX 22.0 KB) [file 11357_2025_1678_MOESM7_ESM.docx]

**Supplementary Table 7.** Associations between gait variables and key-tapping variables in an unadjusted model and a model adjusted for age, sex and years of education.

|  | **Unadjusted Model** | | | **Adjusted Model** | | |
| --- | --- | --- | --- | --- | --- | --- |
|  | β | 95% CI | *P* | β | 95% CI | *P* |
| Gait speed and key-tapping speed (N) | 4.04 | 3.05; 5.02 | **<.001** | 2.97 | 1.94; 4.01 | **<.001** |
| Gait speed and key-tapping speed (D) | 4.00 | 3.04; 4.87 | **<.001** | 3.04 | 2.05; 4.03 | **<.001** |
| Gait frequency and key-tapping frequency (N) | .45 | .28; .62 | **<.001** | .24 | .048; .43 | .015 |
| Gait frequency and key-tapping frequency (D) | .46 | .31; .62 | **<.001** | .28 | .10; .46 | **.002** |
| Gait variability and key-tapping variability (N) | .01 | .00; .01 | **.001** | .004 | .00; .01 | **.008** |
| Gait variability and key-tapping variability (D) | .003 | .00; .01 | **.007** | .003 | .00; .01 | .034 |
| Gait contact and key-tapping contact (N) | .000 | .000; .001 | .028 | .000 | -.0003; .0003 | .849 |
| Gait contact and key-tapping contact (D) | .001 | .000; .001 | **.005** | .000 | -.000; .001 | .164 |

Abbreviations: β, Beta Coefficient; CI, confidence interval; *P,* p value; N, nondominant hand; D, dominant hand.
