# Supplementary material for: Stepping and tapping: combining motor tasks improves cognitive classification
Source: GeroScience. 2025 May 8;48(1):829–42. doi: 10.1007/s11357-025-01678-7 (PMC12972407; doi:10.1007/s11357-025-01678-7)
Supplement: Supplementary file 8 — (DOCX 31.7 KB) [file 11357_2025_1678_MOESM8_ESM.docx]

**Supplementary Table 8.** Classification accuracy of cognitive diagnoses in the adjusted model: Model One (Null Model + a gait variable).

|  | **HC v Dementia** | | | **HC v MCI** | | | **HC v SCI** | | |
| --- | --- | --- | --- | --- | --- | --- | --- | --- | --- |
|  |  | n: 153 |  |  | n: 188 |  |  | n: 140 |  |
|  | AUC | 95% CI | *P* | AUC | 95% CI | *P* | AUC | 95% CI | *P* |
| Null | .89 | .84; .94 |  | .79 | .72; .85 |  | .61 | 50; .71 |  |
| Null + gait speed | .94 | .90; .97 | **.025** | .87 | .82; .92 | **.003** | .69 | .61; .78 | .098 |
| Null + gait frequency | .92 | .87; .96 | .154 | .81 | .75; .87 | .317 | .63 | .54; .73 | .583 |
| Null + gait variability | .91 | .86; .96 | .349 | .82 | .75; .88 | .122 | .62 | .52; .72 | .445 |
| Null + gait contact | .92 | .87; .96 | .151 | .83 | .77; .89 | .097 | .64 | .55; .74 | .455 |
|  |  |  |  |  |  |  |  |  |  |
|  | **SCI v Dementia** | | | **SCI v MCI** | | | **MCI v Dementia** | | |
|  |  | n: 127 |  |  | n: 162 |  |  | n: 175 |  |
|  | AUC | 95% CI | *P* | AUC | 95% CI | *P* | AUC | 95% CI | *P* |
| Null | .87 | .81; .94 |  | .74 | .66; .82 |  | .70 | .62; .78 |  |
| Null + gait speed | .89 | .83; .95 | .285 | .77 | .70; .85 | .197 | .70 | .62; .78 | .903 |
| Null + gait frequency | .88 | .82; .94 | .389 | .75 | .68; .83 | .446 | .69 | .62; .77 | .831 |
| Null + gait variability | .89 | .83; .95 | .287 | .78 | .70; .85 | .140 | .70 | .62; .78 | .142 |
| Null + gait contact | .88 | .82; .94 | .441 | .76 | .69; .84 | .269 | .69 | .62; .77 | .810 |

Area under the Receiver Operating Characteristic curve (AUC) and 95% Confidence Intervals for gait variables in comparison to the Null Model comprising age, sex and years of education. *P* <.05 indicates adding gait variable improves prediction of diagnosis over and above the variables of age, sex and education. Abbreviations: HC, healthy controls; MCI, mild cognitive impairment; SCI, subjective cognitive impairment; AUC, n, number; CI, Confidence Interval; *P,* p-value.
