# Supplementary material for: Stepping and tapping: combining motor tasks improves cognitive classification
Source: GeroScience. 2025 May 8;48(1):829–42. doi: 10.1007/s11357-025-01678-7 (PMC12972407; doi:10.1007/s11357-025-01678-7)
Supplement: Supplementary file 9 — (DOCX 27.5 KB) [file 11357_2025_1678_MOESM9_ESM.docx]

**Supplementary Table 9.** Classification accuracy of cognitive diagnoses in an adjusted model: Model Two comprises Model One (the Null Model plus a gait variable) plus the corresponding key-tapping variable of the **nondominant hand**.

|  | **HC v Dementia** | | | **HC v MCI** | | | **HC v SCI** | | |
| --- | --- | --- | --- | --- | --- | --- | --- | --- | --- |
|  |  | n: 101 |  |  | n: 108 |  |  | n: 97 |  |
|  | AUC | *95% CI* | *P* | AUC | *95% CI* | *P* | AUC | *95% CI* | *P* |
| Null + gait speed AUC | .92 | .87; .97 |  | .81 | .73; .89 |  | .71 | .61; .81 |  |
| Null + gait speed + key-tapping speed | .97 | .94; 1.00 | **.029** | .91 | .86; .97 | **.002** | .74 | .64; .84 | .291 |
| Null + gait frequency | .89 | .82; .95 |  | .71 | .61; .81 |  | .62 | .50; .73 |  |
| Null + gait frequency + key-tapping frequency | .96 | .92: 1.00 | **.027** | .91 | .86; .97 | **<.001** | .69 | .59; .80 | .182 |
| Null + gait variability | .89 | .83; .96 |  | .74 | .65; .84 |  | .62 | .51; .74 |  |
| Null + gait variability + key-tapping variability | .94 | .89; .99 | **.029** | .90 | .85; .96 | **.001** | .71 | .60; .81 | .205 |
| Null + gait contact | .88 | .82; .95 |  | .75 | .66; .84 |  | .68 | .57; .79 |  |
| Null + gait contact + key-tapping contact | .92 | .86; .97 | .066 | .84 | .76; .91 | **.021** | .72 | .62; .83 | .172 |
|  |  |  |  |  |  |  |  |  |  |
|  | **SCI v Dementia** | | | **SCI v MCI** | | | **MCI v Dementia** | | |
|  |  | n: 84 |  |  | n: 91 |  |  | n: 95 |  |
|  | AUC | 95% CI | *P* | AUC | 95% CI | *P* | AUC | 95% CI | *P* |
| Null + gait speed AUC | .87 | .80; .95 |  | .68 | .57; .79 |  | .71 | .61; .82 |  |
| Null + gait speed + key-tapping speed | .91 | .85; .97 | .079 | .78 | .68; .88 | .048 | .76 | .67; .86 | .156 |
| Null + gait frequency | .86 | .78; .94 |  | .66 | .55; .77 |  | .71 | .60; .81 |  |
| Null + gait frequency + key-tapping frequency | .90 | .83; .97 | .132 | .79 | .69; .89 | .021 | .76 | .66; .86 | .207 |
| Null + gait variability | .86 | .78; .94 |  | .73 | .62; .84 |  | .70 | .60; .81 |  |
| Null + gait variability + key-tapping variability | .89 | .82; .96 | .154 | ,76 | .66; .86 | .397 | .75 | .60; .81 | 299 |
| Null + gait contact | .85 | .77; .94 |  | .67 | .55; .78 |  | .70 | .59; .80 |  |
| Null + gait contact + key-tapping contact | .86 | .78; .94 | .296 | .69 | .58; .80 | .281 | .72 | .61; .82 | .500 |

Area under the Receiver Operating Characteristic curve (AUC) and 95% Confidence Intervals for gait variables in comparison to the Null Model comprising age, sex and years of education. *P* <.05 indicates key-tapping variable improves prediction of diagnosis over and above demographic variables. Abbreviations: HC, healthy controls; MCI, mild cognitive impairment; SCI, subjective cognitive impairment; AUC, area under the Receiver Operating Characteristic curve; CI, Confidence Interval; *P,* p-value. ND, nondominant hand; D, dominant hand.
